# Structural Basis for Distinct Binding Properties of the Human Galectins to Thomsen-Friedenreich Antigen

**DOI:** 10.1371/journal.pone.0025007

**Published:** 2011-09-20

**Authors:** Cheng-Feng Bian, Ying Zhang, Hui Sun, De-Feng Li, Da-Cheng Wang

**Affiliations:** 1 National Laboratory of Biomacromolecules, Institute of Biophysics, Chinese Academy of Sciences, Beijing, People's Republic of China; 2 Graduate School of the Chinese Academy of Sciences, Beijing, People's Republic of China; 3 State Key Laboratory of Virology, College of Life Science, Wuhan University, Wuhan, People's Republic of China; Bangor University, United Kingdom

## Abstract

The Thomsen-Friedenreich (TF or T) antigen, Galβ1-3GalNAcα1-*O*-Ser/Thr, is the core 1 structure of O-linked mucin type glycans appearing in tumor-associated glycosylation. The TF antigen occurs in about 90% of human cancer cells and is a potential ligand for the human endogenous galectins. It has been reported that human galectin-1 (Gal-1) and galectin-3 (Gal-3) can perform their cancer-related functions *via* specifically recognizing TF antigen. However, the detailed binding properties have not been clarified and structurally characterized. In this work, first we identified the distinct TF-binding abilities of Gal-1 and Gal-3. The affinity to TF antigen for Gal-3 is two orders of magnitude higher than that for Gal-1. The structures of Gal-3 carbohydrate recognition domain (CRD) complexed with TF antigen and derivatives, TFN and GM1, were then determined. These structures show a unique Glu-water-Arg-water motif-based mode as previously observed in the mushroom galectin AAL. The observation demonstrates that this recognition mode is commonly adopted by TF-binding galectins, either as endogenous or exogenous ones. The detailed structural comparisons between Gal-1 and Gal-3 CRD and mutagenesis experiments reveal that a pentad residue motif (^51^AHGDA^55^) at the loop (g1-L4) connecting β-strands 4 and 5 of Gal-1 produces a serious steric hindrance for TF binding. This motif is the main structural basis for Gal-1 with the low affinity to TF antigen. These findings provide the intrinsic structural elements for regulating the TF-binding activity of Gal-1 in some special conditions and also show certain target and approach for mediating some tumor-related bioactivities of human galectins.

## Introduction

Galectins are a family of lectins, which is characterized by the affinity for recognizing β-galactoside-containing oligosaccharides through the evolutionarily conserved carbohydrate recognition domain (CRD) [1]–[4]. The CRD is constituted of a consensus sequence of about 130 amino acids shared by all members of the family [5]. To date, at least 15 mammalian galectins have been identified [3], which can be further divided into three sub-families according to their general structures [5]. Among them, galectin-1 (Gal-1) and galectin-3 (Gal-3) are widely studied and best characterized.

Galectin-1 is a 14.5 kDa proto-type galectin with one unique CRD, and forms a non-covalent homodimer [4]. Gal-3 is a 31 kDa chimera-type galectin. It is composed of unusual tandem repeats at the N-terminus and CRD domain at the C-terminus [5]. The molecule used in this study is a truncated CRD domain of Gal-3, which will be called as Gal-3 CRD here after. As endogenous galectins, they can exist in the cytoplasm and nucleus through passive diffusion or active transportation [6]–[7], or on the plasma membrane surface as the components of the extracellular matrix [8]. So far, many of the intracellular and extracellular functions of Gal-1 and Gal-3 have been identified [9]–[10]. Among others, cancer-associated activities have been gained much attention. It has been reported that Gal-1 and Gal-3 exert their cancer-related processes through specifically recognizing the Thomsen-Friedenreich (TF) antigen [11]–[15].

TF antigen, Galβ1-3GalNAcα1-*O*-Ser/Thr, is the core 1 structure of O-linked mucin type glycans. The intact structure of TF antigen consists of three moieties: an N-acetyl galactose (GalNAc) linking with a galactose (Gal) and a Ser/Thr residue. It is known that TF antigen occurs in ∼90% of all human cancer cells in cancer and pre-cancerous conditions [16]. It has been reported that cell surface glycoproteins with TF antigen play an important role in the regulation of cancer cell proliferation [17]–[18]. The structures of two galectins, CGL2 and N-terminal domain of galectin-9, complexed with TF disaccharide have been reported [19]–[20], but they did not show any interactions between the galectins and the GalNAc moiety unique for TF antigen. Most recently, the structure of TF antigen complexed with an anti-tumor galectin AAL from the edible mushroom *Agrocybe aegerita* [21] has been reported to provide a first look at the recognition mode between an exogenous galectin and TF antigen, which is unique in a conservative Glu-water-Arg-water structural motif. Though several structures of human Gal-1 [22]–[24] and Gal-3 CRD [25]–[28] with different ligands have been determined, there is no report on the binding properties of the human endogenous galectins with TF antigen based on the structural characterization.

Here we report the identification of the distinct TF-binding properties of Gal-1 and Gal-3 CRD and the crystal structures of Gal-3 CRD complexed with TF antigen and derivatives, TFN (TF p-nitrophenyl) and GM1 pentasaccharide (Galβ1-3GalNAcβ1-4[NeuAcα2-3]Galβ1-4Glc), respectively. The results show a conservative TF-binding mode based on the Glu-water-Arg-water motif for the human endogenous galectins, and also reveal essential differences of TF-binding activities between Gal-1 and Gal-3 CRD and the main structural bases for their distinctions. The mutagenesis further identified the observation based on the structures. It sheds a new insight into the relationship between the endogenous galectins and TF antigen.

## Results and Discussion

### Distinct TF-binding properties for Gal-1 and Gal-3 *in vitro*

**Figure 1:**
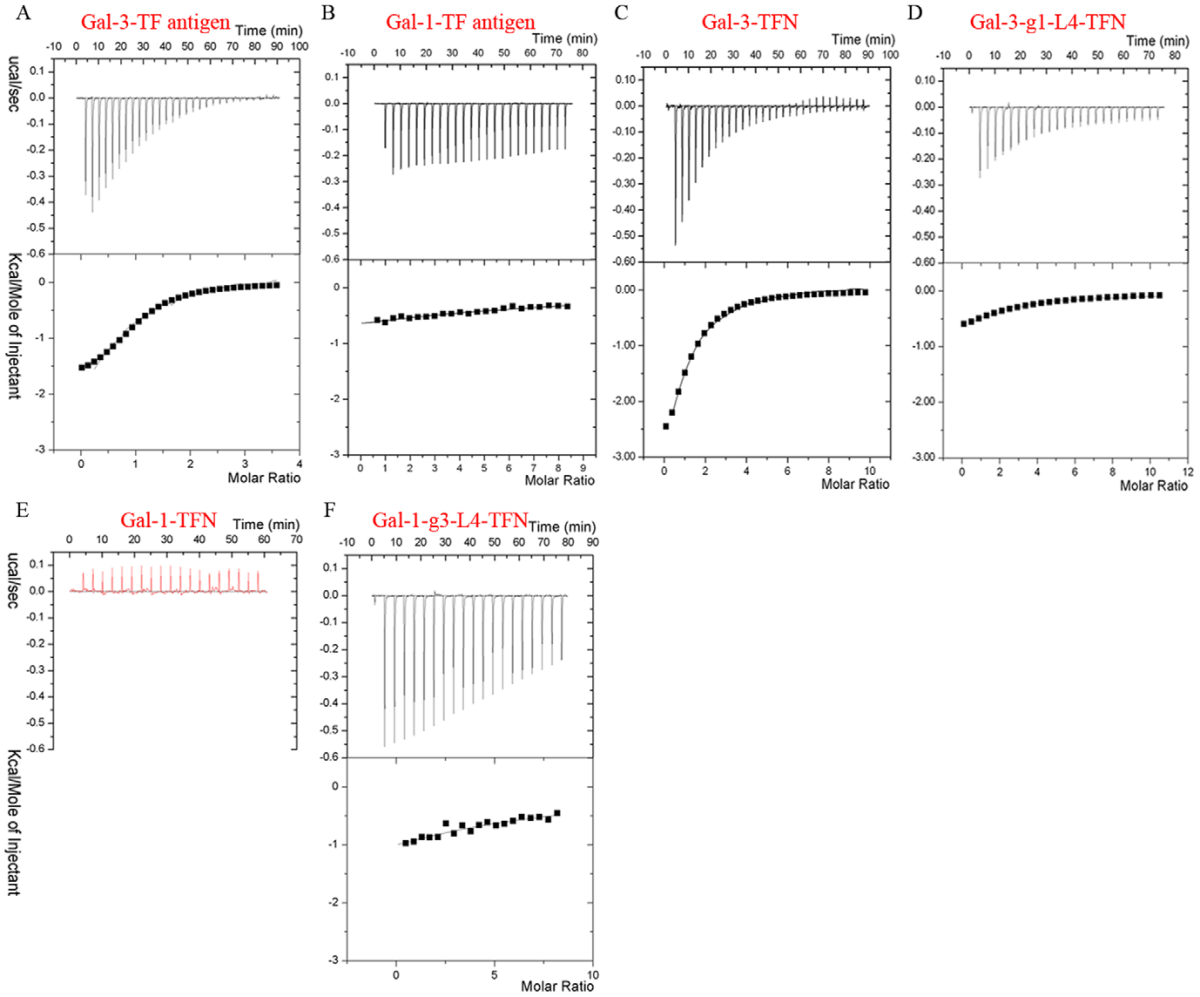
The distinct TF-binding specificities measured by ITC assay at 298 K. ITC measurements using TF antigen titrating native Gal-3 CRD (A) and Gal-1 (B) identify their distinct binding properties. The binding curves of Gal-3 (C) and Gal-3-g1-L4 (D) titrated by TFN show the mutant Gal-3-g1-L4 exhibits very lower affinity to TFN. (E) The red curve of Gal-1 titrated by TFN shows native Gal-1 can not recognize with TFN. (F) The measured curve of Gal-1-g3-L4 titrated by TFN shows the mutant can bind and interact with TFN.

The isothermal titration calorimetry (ITC) assays (Fig. 1A, 1B) show that Gal-1 and Gal-3 CRD possess distinct TF-binding properties. In detail, Gal-3 and Gal-1 bind to TF antigen with dissociation constants (Kd) of 47 µM and 4 mM (Fig. 1A, 1B), respectively, which showed that the affinity to TF antigen for Gal-3 CRD is two orders of magnitude higher than that for Gal-1. Gal-3 also binds to two TF derivatives, TFN and GM1, with Kd of 65 µM (Fig. 1C) and 57 µM (Figure S1), respectively. However, the ITC assays showed that Gal-1 does not bind TFN (Fig. 1E) or GM1 glycan (Figure S1) at all. A parallel assay for Gal-1 binding lactose was performed, which showed that the affinity at Kd = 48 µM was compatible to the previous data [29]. The parameters determined by ITC assay are summarized in Table 1.

**Table 1:** Summary data of measured parameters for Gal-1, -3 and mutants interacting with TF antigen or TF-derivatives from ITC and SPR assays at 298 K.

ITC				SPR
	Kd (**µ**M)	-ΔH (kcal/mol)	ΔS (cal/mol/deg)	N, stochiometry	Kd (**µ**M)
Gal-1- TF antigen	4000	26.3	−77.2	1.00	
Gal-1- TFN	No affinity				
Gal-1- GM1	No affinity				No affinity
Gal-1-g3-L4-TFN	719	1.5	9.4	1.00	
Gal-1-g3-L4-GM1	568	10.3	−19.8	1.00	Low affinity
Gal-3-TF antigen	47	1.9	13.5	1.04	
Gal-3-TFN	65 (100%)	4.6	3.8	1.21	
Gal-3-GM1	57 (100%)	4.0	6.0	1.06	62 (100%)
Gal-3-g1-L4-TFN	543 (12%)	3.9	1.9	1.00	
Gal-3-g1-L4-GM1	415 (14%)	3.2	4.7	1.00	397 (16%)

Errors in Kd range from 4% to 12%. Errors in ΔH are less than 2%. In the assays of TF titrating Gal-1 and two mutants, the stochiometry is fixed with a value of 1; in other measurements, errors in N value are less than 8%. Square distance numbers came from fitting procedure.

**Figure 2:**
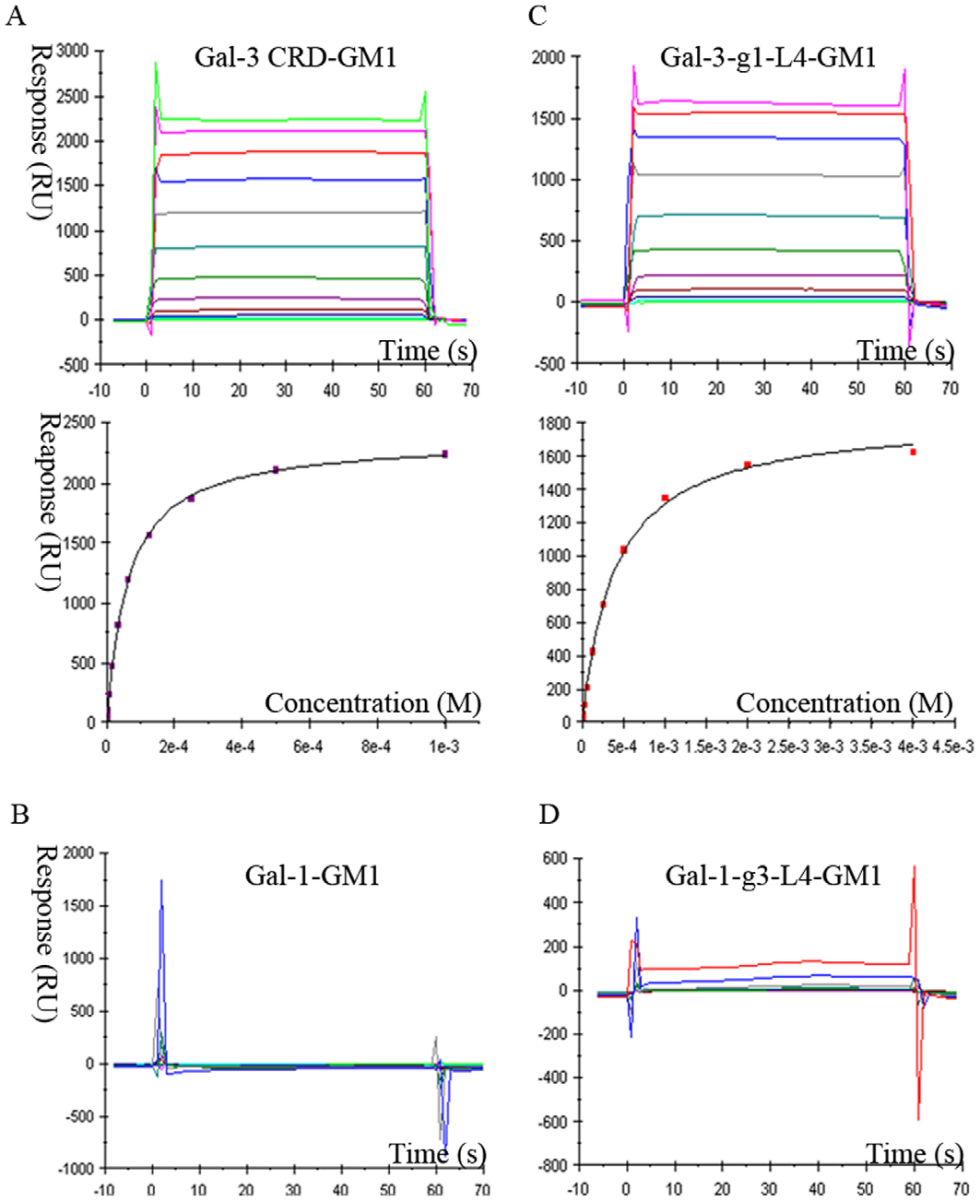
SPR analysis of the binding specificities for Gal-1, -3 CRD and mutants to immobilized GM1-biotin pentasaccharide at 298 K. (A) The binding curves measured at different concentrations of Gal-3 CRD and the steady state affinity to biotin labeled GM1 pentasaccharide. (B) The sensorgram response profiles for Gal-1 at different concentrations show no interaction between Gal-1 and GM1-biotin. (C) The sensorgram curves and steady state analysis illustrate the mutant Gal-3-g1-L4 with lower affinity to GM1-biotin. (D) The measured curves show the mutant Gal-1-g3-L4 acquires the binding ability to GM1-biotin.

The surface plasmon resonance (SPR) assays further confirm TF-containing glycan GM1 can bind to Gal-3, but not Gal-1. The registered curves obtained automatically from different sensorgrams show that Gal-3 CRD can be efficiently captured by the sensor chip surface through binding to the biotin labeled GM1 pentasaccharide and quickly dissociated to the baseline level (Fig. 2A). Analysis of steady state affinity model identified that the Kd is 62 µM, which is identical to the ITC data (Table 1). However, the signal curves referencing of Gal-1 binding with biotin labeled GM1 is negative. In different sensorgrams, analytes of Gal-1 at various increasing concentrations did not interact with GM1 (Fig. 2B).

In a word, both ITC and SPR assays identified that Gal-3, but not Gal-1, binds TF antigen and its derivatives with a relative high affinity, which show that Gal-1 and Gal-3 CRD possess distinct TF-binding properties.

### Crystal structures of Gal-3 CRD complexed with TF antigen and derivatives show a conservative TF-binding mode

Gal-3 CRD in complex with TF antigen, lactose, TFN or GM1 were crystallized and their crystal structures were then determined by using the molecular replacement method with Gal-3 CRD structure (PDB ID 2NN8) [25] as the search model. The crystal structures of Gal-3 CRD in the presence of TF antigen, lactose, TFN or GM1 were determined at 2.0 Å, 2.0 Å, 1.9 Å and 1.8 Å, respectively. The statistics of data collection and structural refinement are summarized in Table 2. However, no complex crystals were obtained in co-crystallization of Gal-1 with TF antigen, TFN or GM1. These further conform that Gal-1 and Gal-3 have distinct binding properties for TF antigen and TF-containing ligands.

**Table 2:** Data collection, processing and structural refinement statistics for Gal-3 CRD complexed with ligands.

Crystal	G3C-lactose	G3C-TF	G3C-TFN	G3C-GM1
PDB code	3AYE	3AYA	3AYD	3AYC
Data collection				
Wavelength (Å)	1.5418	1.5418	0.9793	0.9793
Space group	P1	P1	P3_2_21	P1
Unit cell parameters				
a, b, c (Å)	32.64, 51.67, 60.10	32.72, 51.33, 59.77	50.73, 50.73, 106.78	32.54, 51.64, 59.67
α, β, γ (°)	64.37, 86.25, 80.47	64.70, 85.33, 80.33	90, 90, 120	64.44, 85.23, 81.04
Resolution (Å)	32.19-2.00 (2.11-2.00)	32.25-2.00 (2.11-2.00)	40.62-1.90 (2.00-1.90)	29.28-1.80 (1.90-1.80)
Subunits/asymmetric unit	2	2	1	2
Unique reflections	22,109	21,917	13,046	31,267
Completeness (%)	93.8 (91.1)	93.6 (90.9)	99.4 (98.8)	97.5 (96.3)
Redundancy	2.0 (2.0)	3.9 (3.9)	9.7 (5.7)	3.4 (3.4)
Mean I/σ (I)	12.7 (7.4)	21.7 (13.7)	29.4 (15.4)	10.9 (6.8)
R_merg_ (%)	2.9 (5.8)	4.2 (6.7)	5.8 (7.6)	8.3 (13.7)
Refinement				
R-factor	17.5	19.9	18.2	17.0
R_free_-factor	21.0	23.8	21.7	20.1
Average B-factor for overall, water, ligand (Å^2^)	24.1, 36.7, 31.7	21.6, 36.8, 36.1	23.9, 38.5, 20.8	17.2, 30.7, 19.9
Protein atoms	2172	2172	1086	2172
Water molecules	358	424	245	456
Atoms of ligands	46	66	70	74
RMSDs from ideal model				
Bond (Å)	0.006	0.024	0.007	0.008
Angles (°)	1.5	1.7	1.5	1.8
Ramachandran plots				
Most favored regions (%)	84.1	81.5	84.5	84.1
Allowed regions (%)	15.9	18.5	15.5	15.9
Disallowed regions (%)	0	0	0	0

Values in parentheses are for the highest resolution shell.

**Figure 3:**
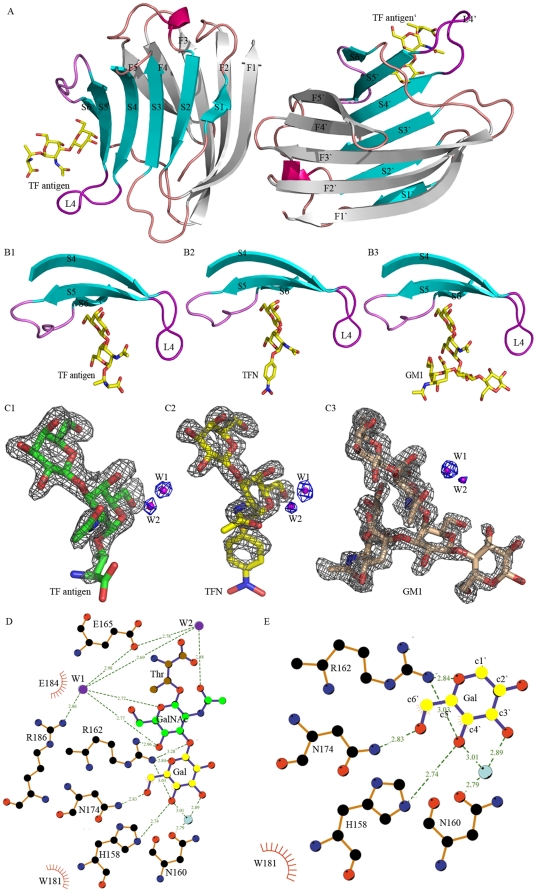
Overall structures of Gal-3 CRD complexed with TF antigen and its derivatives. (A) Two Gal-3 CRD-TF complex molecules in an asymmetry unit. TF antigen bound to the CRD concave is shown in a stick model. (B) The ligands binding site in complexes Gal-3-TF antigen (B1), Gal-3-TFN (B2) and Gal-3-GM1 (B3), which show a conservative location at the concave formed by β-strands S4, S5, S6 and a loop L4 connecting S4, S5. (C) Fo-Fc electron density maps around TF (C1), TFN (C2) and GM1 (C3) with two conservative water molecules (contoured at 3.0 σ). (D, E) Interactions between Gal-3 CRD and TF antigen or TF moiety in TFN and GM1, in which the GalNAc and Gal moieties are shown in green and yellow, respectively.

The general folds of Gal-3 CRD in these complexes show a high similarity to the previously reported structures [25] with Cα atoms root mean square deviation (r.m.s.d) of 0.33 Å, 0.32 Å, 0.45 Å and 0.37 Å, respectively. In the complex structures, there are two Gal-3 CRD monomers binding with glycans in an asymmetric unit (Fig. 3A). The CRD adopts a typical galectin fold in which six-stranded (S1–S6) and five-stranded (F1–F5) antiparallel β-sheets jointly formed a β-sandwich structure. The S1–S6 β-strands constitute a concave surface on which TF antigen and other glycans are bound. All these structural features are like those previously reported [25]–[28]. In the present structures, the residues involving in TF-binding are located on S4–S6 β-strands and the loop connecting S4 and S5 (Fig. 3B). Electron density maps show that TF antigen, TFN and GM1 in the complexes are all well ordered (Fig. 3C) and carbohydrate rings of TFs are in the chair conformations.

As the core 1 structure O-linked mucin type glycan, an intact TF antigen consists of three moieties: an N-acetyl galactose (GalNAc) linking with a galactose (Gal) and a Ser/Thr residue (Galβ1-3GalNAcα1-*O*-Ser/Thr). The moiety of GalNAc is unique for TF antigen. In the structure of Gal-3 CRD-TF complex, TF antigen is bound to the concave pocket formed by S4–S6 β-strands with the insertion of β-galactose ring into a wedge like cavity. The Gal moiety of TF antigen interacts with residues His158, Asn160, Arg162, Asn174, Trp181 and Glu184 *via* the hydrogen bonds or van der waals contacts (Fig. 3E). The recognition way for Gal moiety is basically identical to those observed in other Gal-3 CRD related structures [25]–[28].

**Figure 4:**
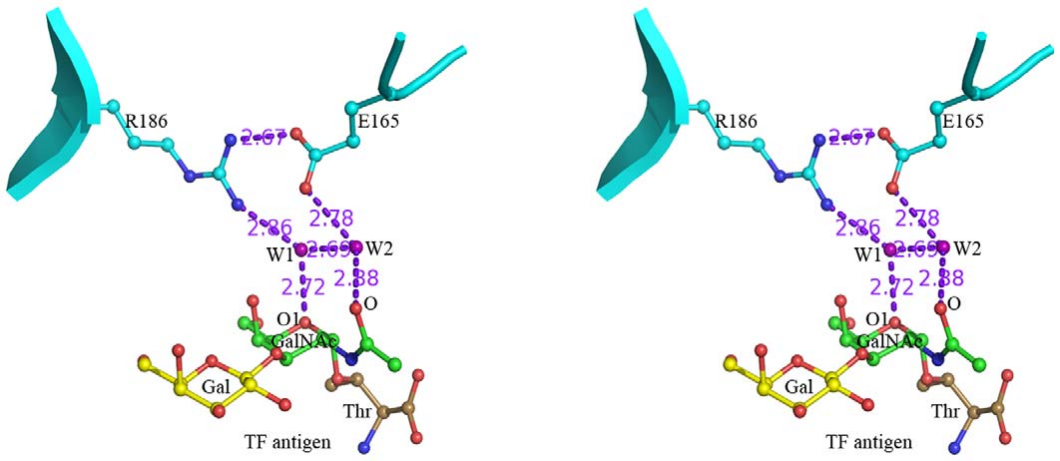
The specific interaction and binding mode in Galectin-TF antigen complex. Stereo diagrams show the specific recognition for human Gal-3 interacting with TF antigen is unique in an Arg186-Water-Glu165-Water motif-based hydrogen bond network. The hydrogen bonds are shown as dashed lines.

**Figure 5:**
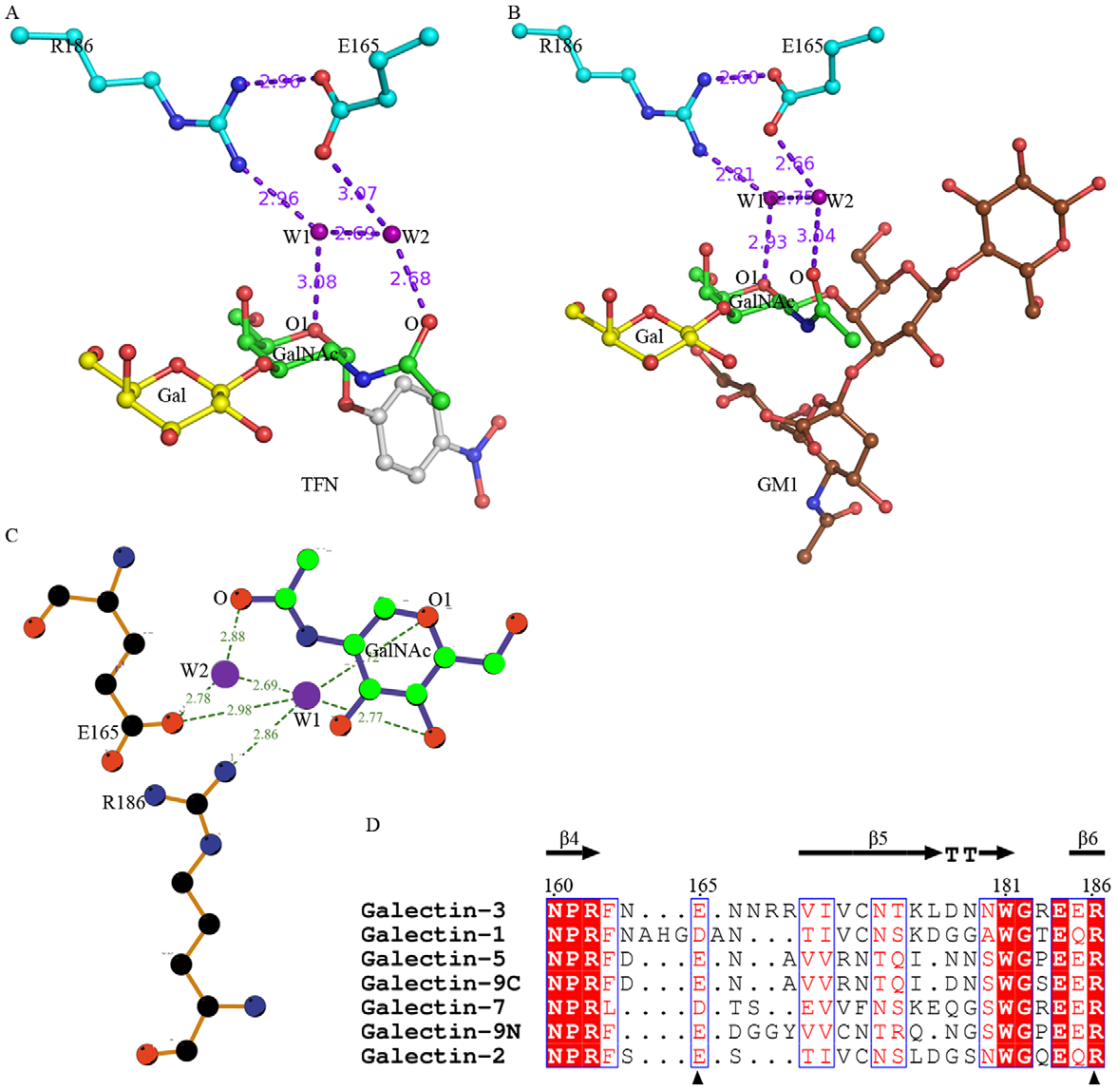
The conservative Arg186-Water-Glu165-Water motif-based TF-binding mode. (A, B) The specific recognition mode is conserved in Gal-3-TFN or Gal-3-GM1 complex. (C) Consensus binding mode between galectins and TF antigen, which is unique in a water molecule mediated hydrogen bond network. (D) Sequence alignment of carbohydrate recognition domain for human galectins. The critical residues for the binding mode highlighted in triangles are conservative.

For the GalNAc moiety of TF antigen, a unique recognition mode is observed, which is consisted of a hydrogen bond network including two water molecules, W1 and W2 (Fig. 4). In this network, residue Arg186 interacts with atom O1 on the carbohydrate ring of GalNAc moiety *via* water molecule W1, while residue Glu165 contributes to recognition with the N-acetyl group through water molecule W2. In addition, these two paths are closely connected with each other *via* the hydrogen bonds between residues Arg186 and Glu165 and two water molecules. The Arg186-water-Glu165-water motif-based network forms a unique paradigm, which was firstly reported in the mushroom galectin AAL-TF complex [21], to specifically recognize TF antigen. In the structures of Gal-3 CRD complexed with TFN or GM1 (Fig. 5A, 5B), the Arg186-water-Glu165-water motif-based hydrogen bond network involving in the recognition with GalNAc moiety of TF disaccharide is very conservative. Gal-3 CRD recognizes the TF disaccharide moiety of GM1, not the lactose moiety as previously supposed [30]. Moreover, in all known structures of galectins complexed with non-TF ligands, there is no such unique recognition mode to be found. The structure-based sequence alignments of the galectins potentially interacting with TF antigen show that the two residues Arg and Glu essential for TF-recognition are also conservative (Fig. 5D). These observations cooperatively show that the specific recognition mode should be commonly adopted by TF-binding galectins, either as endogenous or exogenous ones.

### The structural basis for distinct TF-binding property of Gal-1

**Figure 6:**
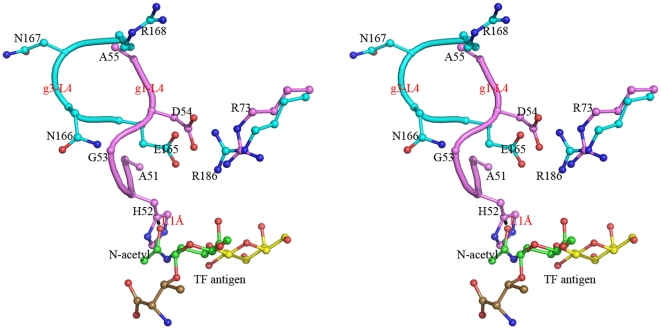
Structural superposition and comparison between the two loops, g1-L4 and g3-L4. Stereo view of the loops g1-L4 (in pink) and g3-L4 (in cyan) show their different conformations. The residue His52 on the loop g1-L4 make close contacts with the N-acetyl group of TF antigen with a distance of 1.1 Å between atoms C_γ_ of His52 and O of the N-acetyl group.

**Figure 7:**
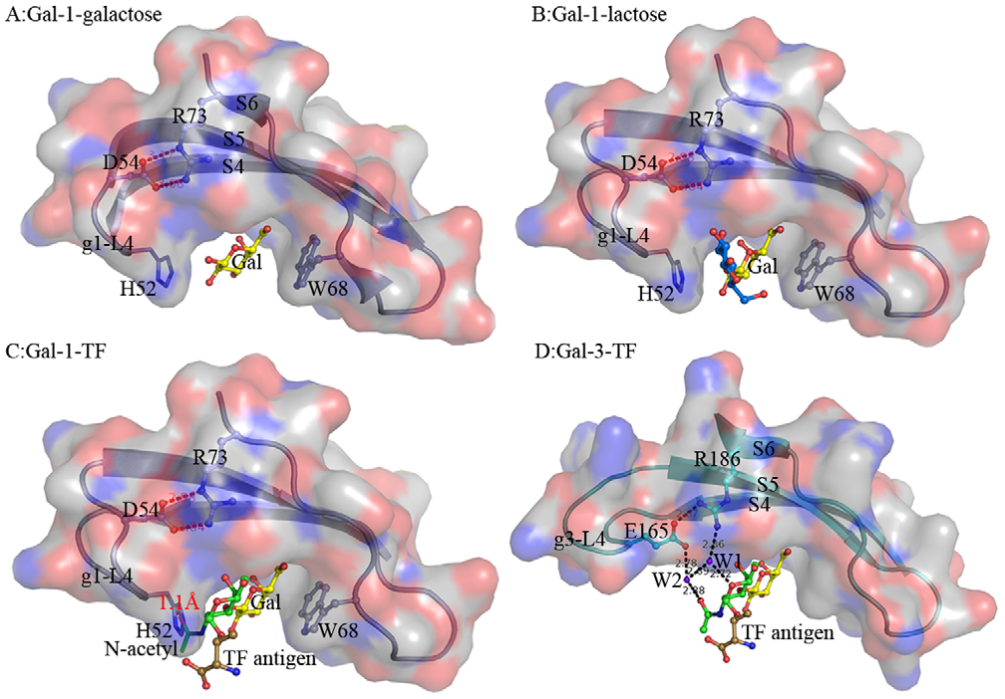
Structural comparison of the carbohydrate recognition pockets in Gal-1 and Gal-3 binding with different ligands. (A, B) The concave carbohydrate binding pocket in Gal-1 shows Gal-1 recognizes and binds with galactose (Gal) or alternative lactose suitably. (C) The modeled TF antigen shows a serious spatial obstruction between its N-acetyl group and His52 on g1-L4. (D) The TF-binding pocket and the unique binding mode conserved in Gal-3 CRD. All ligands are shown in a ball-and-stick model and carbohydrate binding pockets are illustrated as surface drawing.

Structural superimpositions of Gal-1 structures (PDB ID 1W6N, 1W6M and 1GZW [22]) with Gal-3 CRD-TF antigen complex structure show that the general folds of their CRD domains, including the main TF-binding location consisted of β-strands S4, S5 and S6, are very similar. However, it is interesting to find that the loop (L4) between S4 and S5 takes evidently different conformation and stereo-chemistry in Gal-1 and Gal-3 (Fig. 6). Loop L4 of Gal-1 (g1-L4) consists of a pentad residue motif, ^51^AHGDA^55^, whereas the corresponding one (g3-L4) of Gal-3 constitutes with a tetrad residue motif, ^165^ENNR^168^. In turn, loop g1-L4 of Gal-1 is folded rather inner so that the relative cavity is narrower for TF antigen binding. The essentially restricted factor for TF binding comes from the unique residue, His52. After modeling a GalNAc group unique for TF antigen instead of the glucose moiety in Gal-1-lactose complex structure, the atom O of N-acetyl group makes close contacts with the imidazole ring of residue His52 by a distance of 1.11 Å (Fig. 6). Evidently, the steric hindrance of residue His52 appeared at loop g1-L4 would block Gal-1 from binding with TF-containing ligands and push them away. Besides, residue His52 lies on an II-type β-turn, which is stabilized by the hydrogen bond between the carbonyl group of residue Ala51 and the amino group of residue Asp54. Most importantly, residue His52 is one of major residues contributing to binding the β-Gal moiety. It participates in the stacking contacts with the atoms C2′, O2′ and O4 bridging galactose and glucose. It offers van der waals interactions on one face. Residue Trp68 provides the stacking contacts with atoms C3′, C4′, and C5′ on the other face. Both His52 and Trp68 together align the β-Gal moiety. This is a common way for Gal-1 recognizing the β-galacto-glycans in different species [22]–[24], [31]–[32]. Therefore, the conformation of His52 is definite in Gal-1 structure. All together described above cooperatively make the stable conformation and orientation of residue His52 at loop g1-L4 as a unique intrinsic structural characteristic for Gal-1. Gal-1 can bind with β-galactose or lactose ligand (Fig. 7A, 7B), but filtrate out the TF-containing glycans (Fig. 7C). Correspondingly, locations and orientations of the residues Asp54 and Arg73 critical for TF-binding are also remarkably changed in Gal-1, in turn to destroying the conservative TF-binding mode as observed in Gal-3 (Fig. 7D).

### Mutagenesis analysis identified the intrinsic structural element for low TF-binding ability of Gal-1

To identified the main structural elements for TF-binding ability, the mutants of Gal-1 and Gal-3 CRD with inter exchanged loop L4 motifs were constructed and named as Gal-1-g3-L4 (Gal-1-^51^ENNR^54^) and Gal-3-gl-L4 (Gal-3 CRD-^165^AHGDA^169^), respectively. ITC assays show that mutant protein Gal-1-g3-L4, excluding residue His52, dramatically acquires the binding abilities to TFN and GM1 pentasaccharide with Kd of 719 µM (Fig. 1F) and 568 µM (Figure S1D), respectively. SPR assays also confirm that Gal-1-g3-L4 can bind to biotin-labeled GM1 (Fig. 2D). Compared with the native Gal-1, this is a qualitative leap. On the other hand, ITC assays show very low affinity to TF derivatives for mutant Gal-3-g1-L4 (Fig. 1D, S1B). The dissociation constants (Kd) of Gal-3-g1-L4 to TFN and GM1 are 543 µM and 415 µM, respectively (Table 1), which show that affinities of the mutant protein to TFN and GM1 are respectively reduced to 12% and 14% compared to the native Gal-3. Meanwhile, SPR assays (Fig. 2C) show the affinity of mutant Gal-3-g1-L4 to biotin-labeled GM1 is decreased to 16% compared to the native Gal-3. These observations from the mutagenesis identify that the L4 motifs are the key intrinsic structural elements for distinct TF-binding abilities between Gal-1 and Gal-3.

It is also noticed that mutant Gal-3-g1-L4 still keeps certain TF-binding abilities to TFN and GM1, which are even rather higher than that of mutant Gal-1-g3-L4. Sequence alignments and structural superposition reveal that residues located closely to the loop L4 are not identical in Gal-1 and Gal-3. The differences of local sequences and structures would affect the conformation of the loop g1-L4 so as to release the steric hindrance raised from His52 somewhat. Therefore, for mutant Gal-3-gl-L4, the loop g1-L4 could partly but not fully block TF from binding. Besides, the fluorescence polarization assay showed that Gal-3 had a specific affinity to galactose moiety in some galactose-containing ligands [33]–[34], which may also occur in mutant Gal-3-g1-L4 so as to contribute to its remained affinities to TFN and GM1. In case of Gal-1, there are van der waals interactions between the side chain of residue His52 and the Gal moiety of lactose as described above. The mutation of Gal-1-g3-L4, excluding residue His52, will reduce the binding affinities to galactose and lactose. ITC assays illustrate that the Kd of Gal-1-g3-L4 to lactose is 298 µM, which confirm that mutant Gal-1-g3-L4 shows a very lower affinity to lactose. Thus, though mutant Gal-1-g3-L4 gains the binding ability to TFN or GM1, the affinity appears at a low level.

It has been reported that Gal-1 could exert its cancer-related functions through the specific recognition with TF antigen. Our findings may provide a special way by which TF antigen could be recognized *via* modification of the pentad motif (^51^AHGDA^55^) to trigger a conformational change of loop g1-L4 to that as g3-L4 of Gal-3, so as to render the TF-binding ability of Gal-1, *in vivo*.

### Conclusion Remarks

In this study, we firstly identified that Gal-1 and Gal-3 possess distinct TF-binding abilities. The structures of Gal-3 CRD complexed with TF antigen and its derivatives show a unique binding mode based on the Glu-water-Arg-water motif for TF-recongnition as observed in the mushroom antitumor galectin AAL. The structural comparisons and sequence alignments show that the unique TF-binding mode observed in Gal-3 CRD-TF complex should be commonly adopted in TF-binding galectins, either as endogenous or exogenous ones. The detailed structural analyses and following mutagenesis experiments identify a pentad residue motif (^51^AHGDA^55^) of the g1-L4 loop for the low TF-binding affinity of Gal-1, which prompts the intrinsic structural elements for regulating the TF-binding activities of Gal-1 and Gal-3. The findings provide a new target and approach for mediating the tumor-related activities of human galectins and shed lights on the possible way, by which the TF-binding ability of Gal-1 could be regulated under special conditions.

## Materials and Methods

### Purification and crystallization

The genes of Gal-1 and Gal-3 CRD were cloned on the pET-22b vector without a His-tag. The recombinant plasmids were transformed into Escherichia coli strain BL21 (DE3) for expression. The proteins were purified on a lactosyl Sepharose column essentially as described previously [4], and then on a Hiload Superdex75 16/60 column (from GE Healthcare) to remove the lactose. The purified proteins were concentrated to 30 mg/ml in solution buffer containing 10 mM PBS, 100 mM NaCl and 8 mM β-Mercaptoethanol.

The mutants, Gal-1-g3-L4 and Gal-3-g1-L4, were designed by exchanging two loops, g1-L4 and g3-L4. These mutants were constructed using Quick Change Site Directed Mutagenesis method, and then the proteins were expressed and purified as above procedures.

Crystals of Gal-3 CRD binding ligands were obtained from drops containing equal volumes of the protein solution buffer with 10 mM TF antigen (a gift from the CFG), TFN (from Merk) or GM1 pentasaccharide (from Santa cruz) and reservoir buffer composed of 2 M Ammonium sulfate, 0.1 M Bis-Tris pH5.5–6.0, 8 mM β-Mercaptoethanol, using the hanging drop vapor diffusion method at room temperature. Crystals of Gal-3 CRD in complex with TFN generally appeared after 6 hours and grew to full sizes in 1–2 d. Crystals of Gal-3 CRD complexed with TF antigen or GM1 pentasaccharide were acquired through the micro-seeding method.

### Data collection, structural solution and refinement

Crystals of Gal-3 CRD complex with ligands were soaked into the reservoir solution supplemented with 10% (v/v) glycerol as cryo-protectant for 15–20 s, and then flash cryo-frozen to 85 K for data collection. Data of Gal-3 CRD-TF and Gal-3 CRD-lactose were collected on the R-Axis IV++ Image Plate detector at the wavelength of 1.5418 Å using Rigaku rotating anode X-ray generator in house. Data of Gal-3 CRD-TFN and Gal-3 CRD-GM1 were collected at the wavelength of 0.9793 Å on Beamline 17 U synchrotron, Shanghai Synchrotron Radiation Facility. Exposure time per image was 180 s in house or 0.8 s on synchrotron.

The dada were integrated using the program MOSFLM [35] and then scaled by SCALA from CCP4 package [36]. Structures were determined using Phaser [37] with the molecular replacement method using the structure of Gal-3 CRD (PDB ID 2NN8) [25] as the search model. The ligands were manually added and adjusted according to the remnants of electron density maps in Coot [38]. The final refinements of structures were performed using CNS [35] and manual adjustment alternatively. 5% of the reflections for R_free_ calculations were randomly chosen and excluded from every refinement.

### ITC assay

All calorimetric experiments were performed by the ITC200 isothermal titration calorimeter (Microcal, 200 µl cell, Northampton, GE Healthcare, America) at 298 K using 40 µl ligands titrating 200 µl galectins. The delayed time between injections is 180 s. For every ligands, at least three titrations were done. The dissociation constants (Kd) were calculated using the Origin Program and the experimental data were fitted to a one-set-of-sites model.

### SPR assay

The SPR assays were performed at 298 K using the BIAcore 3000 instrumentation (from GE Healthcare, Sweden). Biotin labeled GM1 pentasaccharide (from CFG) was dissolved into PBS buffer and connected covalently onto a streptavidin (SA) chip as the immobilized phase. The fix amount of GM1 is up to 190 response units. Galectins in the sample buffer (20 mM PBS, pH7.5, 0.005% Tween 20) were as the flowing phase. Analytes with volume of 30 µl at various concentrations ordered from low to high were programmed to inject with a 30 µl/min flow rate under different sensorgrams. In order to correct for instrumental and concentration effects, a blank flow channel was used as reference. Specific binding analyses were identified by subtraction of the control channel. The kinetic analyses of molecular interactions were calculated according to the steady state affinity model by BIAcore T100 Evaluation software.

### Protein Data Bank accession number

Coordinates and structure factors for the structures of Gal-3 CRD complexed with TF antigen, TFN and GM1 have been deposited at the Protein Data Bank with accession codes 3AYA, 3AYD, 3AYC, respectively.

## Supporting Information

Figure S1 **ITC measurements of Gal-3 CRD, Gal-1 and two mutants titrated by GM1 pentasaccharide at 298K.** Titrating curves of Gal-3 (A) and Gal-3-g1-L4 (B) show the different affinities to GM1 pentasaccharide. (C) The red curve shows native Gal-1 can not recognize and interact with GM1. (D) The black curve of Gal-1-g3-L4 titrated by GM1 shows the mutant gains the TF-binding ability.
(TIF)
