# Supplementary figures and images for: Structural Basis for Distinct Binding Properties of the Human Galectins to Thomsen-Friedenreich Antigen
Source: PLoS One. 2011 Sep 20;6(9):e25007. doi: 10.1371/journal.pone.0025007 (PMC3176802; doi:10.1371/journal.pone.0025007)

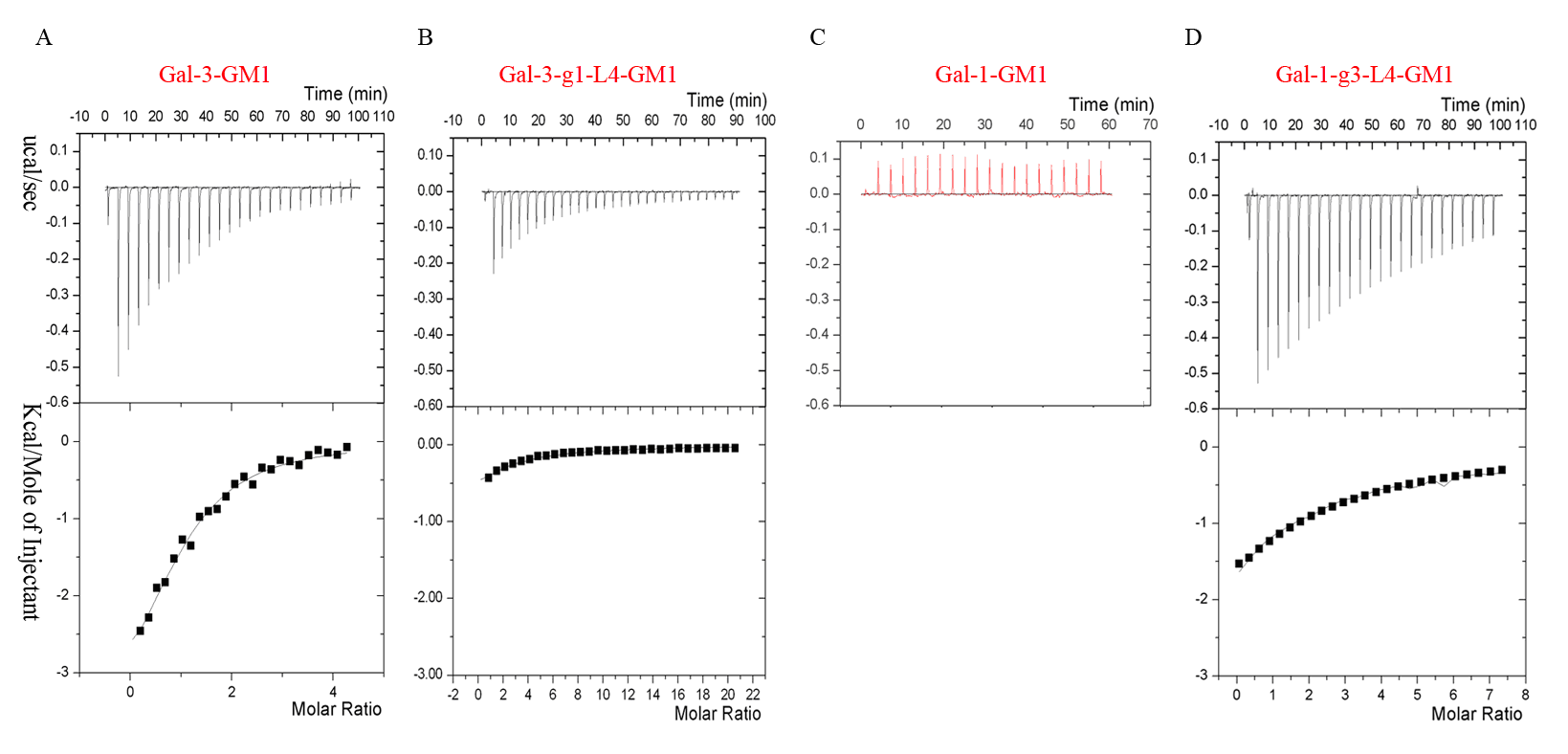

Supplement: Figure S1 — ITC measurements of Gal-3 CRD, Gal-1 and two mutants titrated by GM1 pentasaccharide at 298K. Titrating curves of Gal-3 (A) and Gal-3-g1-L4 (B) show the different affinities to GM1 pentasaccharide. (C) The red curve shows native Gal-1 can not recognize and interact with GM1. (D) The black curve of Gal-1-g3-L4 titrated by GM1 shows the mutant gains the TF-binding ability. (TIF) [file pone.0025007.s001.tif]
